# Research Hotspots and Trends of Exercise on Parkinson's Disease: A Global Bibliometric Analysis From 2012 to 2021

**DOI:** 10.3389/fnhum.2022.908049

**Published:** 2022-05-27

**Authors:** Ji-Wei Chen, Shu-Hao Du, Tian-Cong Chen, Kun Zhu

**Affiliations:** ^1^Department of Physical Education and Sport Training, Shanghai University of Sport, Shanghai, China; ^2^Department of Sport Rehabilitation, Shanghai University of Sport, Shanghai, China; ^3^Department of Rehabilitation Medicine, Shanghai Shangti Orthopaedic Hospital, Shanghai, China

**Keywords:** exercise, Parkinson's, bibliometric analysis, visual analysis, hotspot, trend

## Abstract

**Background:**

Parkinson's disease is a chronic neurodegenerative disease, which can be alleviated in drug treatment, but with evident side effects. At the same time, increasing evidence shows that exercise can significantly improve the symptoms of patients with Parkinson's disease, with an effect that cannot be achieved by drug treatment. The related research on exercise on Parkinson's disease increases rapidly with the passage of time. However, the research analysis on Parkinson's disease by means of bibliometrics is rare. The purpose of this study is to perform a bibliometric analysis of the research hotspots and development trends of the global movement on Parkinson's disease from 2012 to 2021.

**Methods:**

The literature was derived from the Web of Science core collection database, and the social science citation index was set as SCI-EXPANDED. The language was set to English, and the literature category was set as article and review and published from 2012 to 2021. CiteSpace and other software were used to analyze the relationship among published documents, countries, institutions, journals, authors, references, disciplines, and keywords.

**Results:**

A total of 2,222 articles were included in the analysis. The analysis showed that the publication volume increased with the increase in years, with a total of 76 countries and 546 academic journals published; the largest number was that of the United States. The journals are mainly concentrated in the fields of neurology, sports, and ophthalmology. Rush University and *Movement Disorders* journals are the main institutions and journals. The cited keywords show that trial, cognition, and interference are the research hotspots and development trends in recent years.

**Conclusion:**

The number of published articles on Parkinson's disease by exercise has increased rapidly in the past 10 years, and the bibliometric analysis can provide useful information for future research teams and researchers.

## Introduction

Parkinson's disease (PD) is one of the most common neurodegenerative diseases (Pringsheim et al., 2014); the older patients are more likely to be affected, and the patients are characterized by physical stiffness, tremor, bradykinesia, cognitive impairment, and poor dynamic balance (Tangen et al., 2017). According to The Global Burden of Disease Study's assessment, 6.2 million people suffer from PD worldwide (Dorsey and Bloem, 2018). The number of people affected by PD reaches 14.2 million in 2040 (van der Kolk et al., 2018). The prevalence of PD mainly affects the elderly, and the prevalence rate of PD increases with age. The prevalence rate of PD increases gradually in people over 40 years old and reaches a peak between 70 and 79 years old. The prevalence rate of PD in people aged 70–79 in Asia is lower than that in Europe, North America, and Australia (Pringsheim et al., 2014).

As a complex neurodegenerative disease, PD is characterized by the loss of motor and non-motor signs. Drug therapy can relieve these symptoms to some extent, but it will be accompanied by some side effects (van der Kolk et al., 2018). According to the evidence, exercise plays a good role in neuroplasticity and brain self-repair ability (Smith and Zigmond, 2003). In exercise-based treatment, most patients with PD need to deal with gait, posture control, balance, and other physical movement disorders. Drug therapy is usually ineffective for these disorders (Poewe et al., 2017). The level of physical activity in patients with Parkinson's decreases faster than that in healthy people of the same age (Fertl et al., 1993), and physical activity decreases with age, also leading to a decrease in functional level (Morris, 2000). In the treatment of PD, exercise has been proven to delay the deterioration of body motor function and the independent prolongation of physical function (Goodwin et al., 2008; Dibble et al., 2009).

In the exercise intervention with non-drug treatment, Parkinson's patients improved their daily activities and physical exercise ability to a certain extent; they also improved in non-exercise areas, such as prevention of depressive symptoms, executive function, language, and other related areas (Shulman et al., 2013; Cugusi et al., 2015; Altmann et al., 2016; Ferraz et al., 2018; Marusiak et al., 2019). Patients with PD have a higher risk of falls due to a serious decline in balance ability. During tai chi training, the function, posture control, and balance disorder of patients with PD were improved, and the fall risk of patients with PD was reduced (Li et al., 2012).

Bibliometric analysis (Bibliometric analysis) is a quantitative statistical method for the study of published literature (Wang et al., 2020). At present, the research on exercise on PD has increased but almost no targeted bibliometric analysis of exercise. Therefore, the purpose of this study is to use CiteSpace software, bibliometrics analysis, summed up the progress and trend of PD research in the past 10 years, including the number of articles, references, countries, institutions, and keywords to provide a theoretical and practical reference for future researchers and research work.

## Methods

### Source and Search Strategy

The corecollection database in the Web of Science database is used as the source for retrieval, the social science citation index is set to SCI-EXPANDED, and the language is set to English. Some studies have pointed out that Web of Science databases provide more material in the analysis of past literature (Yeung, 2019) and have higher accuracy in journal classification compared to Scopus databases (Wang and Waltman, 2016). Enter TI = Parkinson's OR Parkinsonism OR Parkinson in the title word column and record it as # 1. Enter exercise OR train OR training OR movement OR activity OR activities OR strength OR endurance OR resistance OR stability OR walk OR tai chi OR yoga OR motor control OR core control OR stretch OR run OR muscle energy technique OR Pilates OR hydrotherapy OR water sports OR kinesiotherapy in the title word column and record as # 2. Finally, the combination of # 1 and # 2 is set to “AND,” and the document type is selected as “Articles and Review Articles,” Timespan = 2012.01.01–2021.12.31. Finally, 2,222 articles are obtained, and the final date of retrieval and download is 2021.12.31.

### Analysis Tools

CiteSpace software, a Java application program, is usually used for network visualization and analysis. It has the function of generating a visual knowledge graphs of countries, research institutions, references, keywords, and authors. The software detects the development hotspots and trends of visual literature through database search and progressive visualization in the field of knowledge and can be reflected in highly relevant citations and hot topics by a visual atlas (Synnestvedt et al., 2005). Visual knowledge graph is composed of nodes and links. Different nodes represent research institutions, countries, references, keywords, and other elements, and the cooperative relationship of each part is reflected by link; different years are represented by different colors. Nodes with high centrality are regarded as key points or turning points in a certain field (Pei et al., 2019).

CiteSpace parameter settings: In the CiteSpace software interface, in the Time Slicing column time settings 2012.01–2021.12, each year is a time slice. Pruning selects the critical path Pathfinder option to make the nodes and connections in the network clearer, and Pruning Sliced Networks prunes the network for each time period to make the graphics clearer and more focused.

CiteSpace centrality settings: Enter the visualization interface after setting parameters in CiteSpace, and select Compute Node Centrality in the Nodes option to generate the visualization map.

CiteSpace citation burst settings: Enter the visualization interface after setting parameters in CiteSpace, and generate the visualization interface by selecting View in the Burstness option in the Control Panel interface.

Microsoft Excel is used to chart the literature searched in the Web of Science core database, create trend charts for publication volume and citations, and rank countries, institutions, disciplines, and authors.

### Data Extraction

After the design of the retrieval strategy, the literature and bibliometrics indicators are extracted, and the published literature is downloaded from the Web of Science core database. The number of published articles (countries, institutions, journals, and authors), references, disciplines and keywords, and development trends are extracted using CiteSpace and Microsoft Excel software. The extracted literature data are counted and analyzed in the analysis software, and visual maps and intuitive charts are developed. This study explains the impact of exercise on PD through the following parts: (1) the distribution and development trend is analyzed according to year, country, discipline, institution, and author; (2) the cooperation among countries, institutions, and authors is analyzed; (3) through cluster analysis, citation analysis, and H-index analysis in this field, the citation frequency is the sum of the cited times of all items in a set, which can generally reflect the quality of publications. H-index is the number of papers whose citations are larger than H. This new method is used to evaluate academic achievements. H-index can accurately reflect the academic achievements of researchers and can be used as an index to measure the output of scientific research achievements of researchers (Hirsch, 2005). (4) The hot spots of citations and keywords are analyzed.

## Results

### Forecast and Analysis of Annual Publishing Volume and Growth Trend

**Figure 1:**
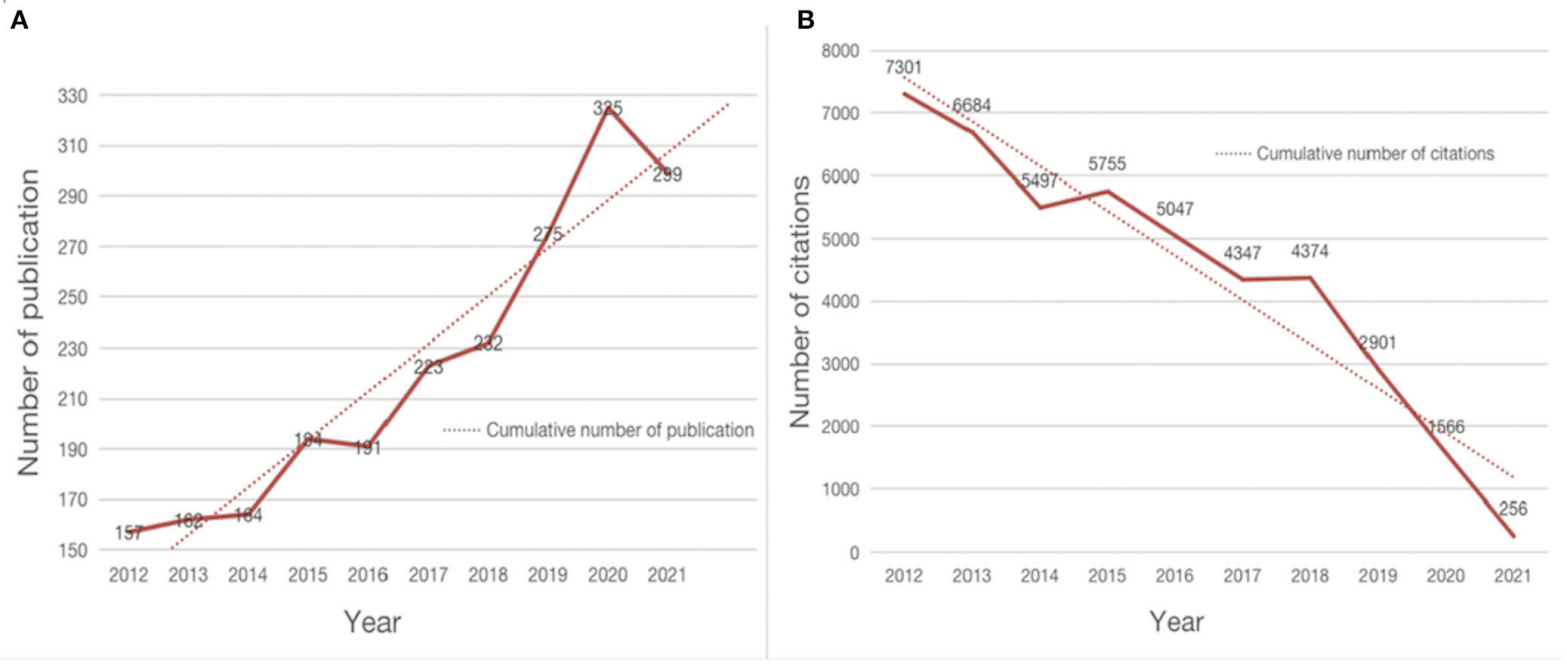
Number of published papers and citations of the exercise on PD research. **(A)** Annual number of publications and trends in exercise on PD research from 2012 to 2021. **(B)** Annual number of citations and trends in exercise on PD research from 2012 to 2021.

A total of 2,222 articles have been included, as shown in Figure 1A. The volume of publications has increased in the past decade, and some fluctuations in the volume of publications have been observed over the past decade. However, the overall trend shows an upward trend with the increase in the number of years. Three stages of rapid growth and one stage of rapid decline have been found in the past decade. It increased from 164 in 2014 to 194 in 2015 (the first rapid growth phase), decreased to 191 from 2015 to 2016, and increased from 191 to 232 between 2016 and 2018 (the second rapid growth phase). Then, it increased from 232 in 2018 to 325 in 2020, reaching the peak of publication in nearly a decade (the third rapid growth phase) in 2020. Subsequently, it declined rapidly from 325 to 299 from 2020 to 2021. However, the overall trend of publishing volume has shown a gradual upward trend in the past 10 years. The number of literature published in the past decade shows that increasing attention has been provided to the research on PD by exercise, and the research on PD through exercise therapy is further carried out.

As shown in Figure 1B, although the number of papers published has increased year by year in the past decade, the number of citations has shown a downward trend due to time factors, and the number of citations has reached the highest in the past decade (7,301) in 2012 because of the longest year.

**Figure 2:**
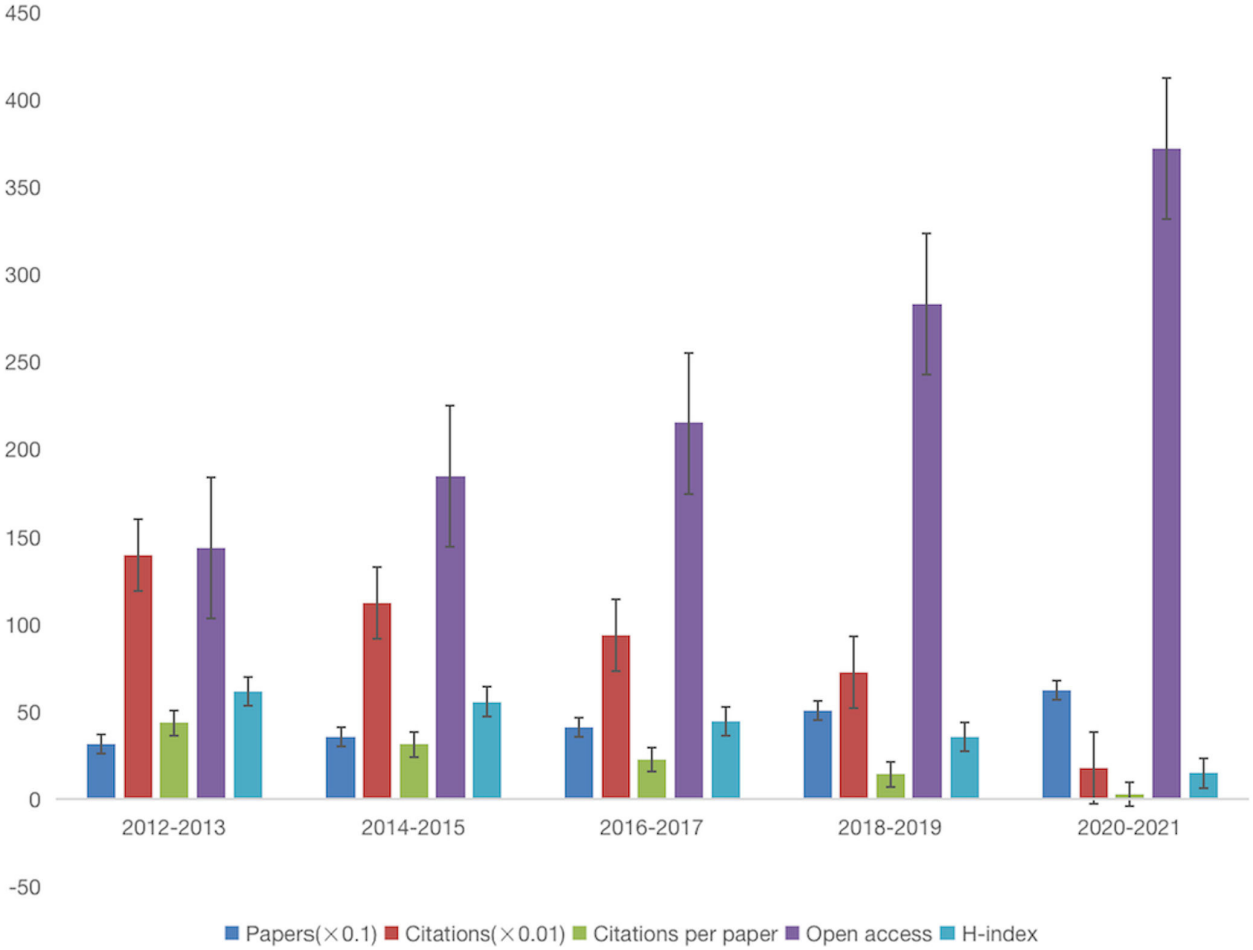
Number of papers, citations, citations per paper, open access, and H-index per 2-year time period for the exercise research on PD.

As shown in Figure 2, among the five-time periods from 2012 to 2021, the number of citations (13,986), the number of citations per paper (43.84), and the H index (62) are the highest, and the number of papers published from 2020 to 2021 (624) and Open access (372) are the highest. In the past 10 years, the number of citations, the number of citations per paper, and H index showed a downward trend due to years. However, the number of open access increased year by year, indicating that with the passage of time, the number of open access increased. This condition was more conducive to the spread of papers, and to a certain extent increased the citation rate of papers.

### Subject Categories of Web of Science

**Figure 3:**
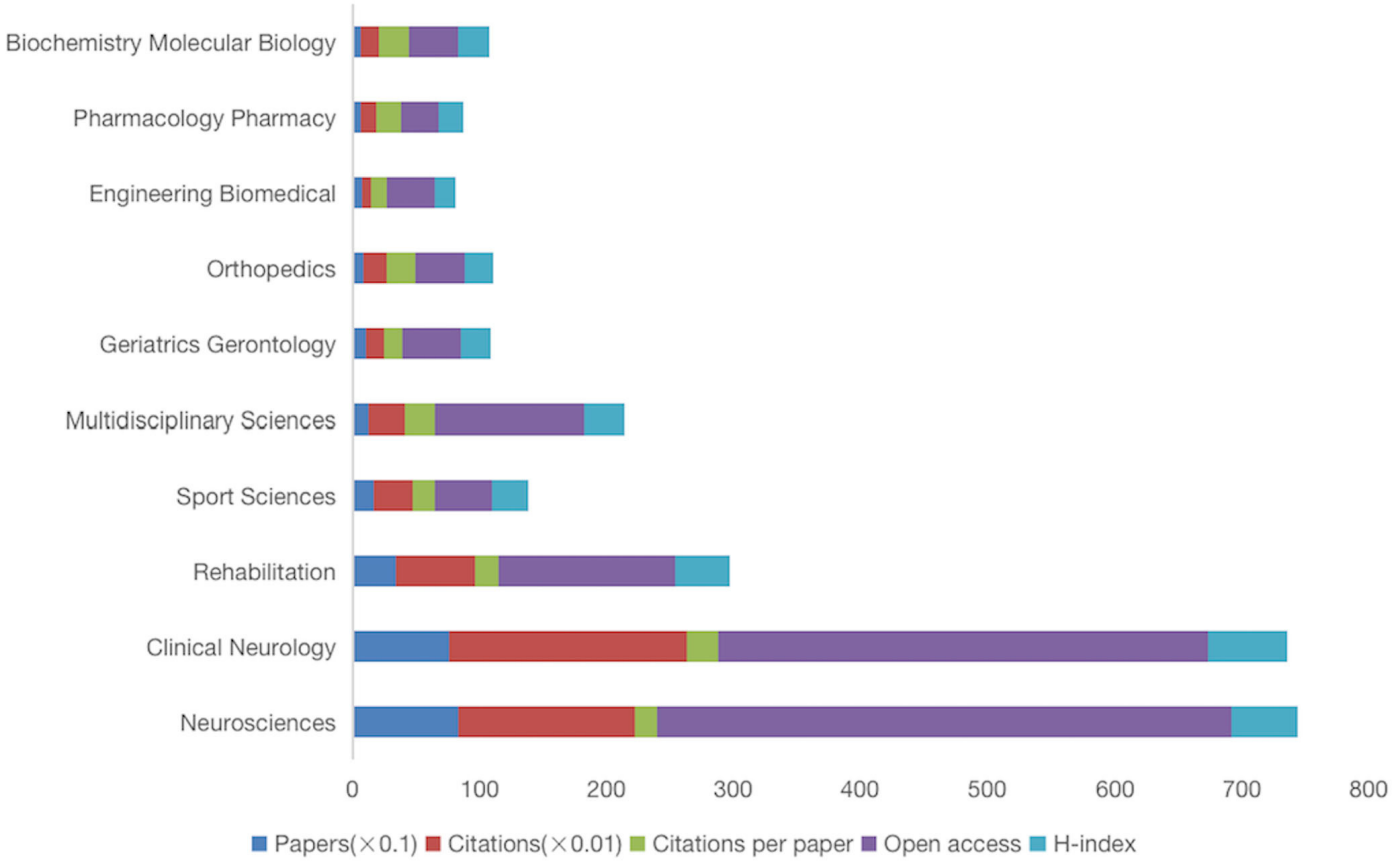
Ranking of the top 10 disciplines related to exercise for PD.

Disciplines were involved in all 2,222 papers on exercise intervention on PD, and we analyzed the top 10 published disciplines (Figure 3). The neurosciences discipline has the largest number of papers published (825) and the largest number of open access (452). Clinical neurology has the largest number of citations (18,781), per article (24.84), and H index (62). In the ranking of disciplines, the publication is mainly concentrated in Neurosciences, Clinical Neurology, Rehabilitation, Sport Sciences, and Multidisciplinary Sciences disciplines.

### Analysis of Document Type

In this study, only papers and reviews are involved. Among which, 1,989 papers account for the largest proportion of 89.514%, and 233 reviews account for 10.486%. An article called “Tai chi and postural stability in patients with PD,” this article is one of the first three most cited, published in the journal *New England Journal of Medicine* (impact Factor 91.253 in 2020). Patients with Parkinson's are randomly assigned to Taijiquan for resistance training and stretching exercises according to the degree of disease, after 24 weeks of intervention twice a week. Compared with resistance training and stretching exercise, Taijiquan can significantly improve balance, posture control, and other functions of patients with PD and effectively reduce the incidence of falls in patients with PD (Li et al., 2012). The most frequently cited article in the review is “Exercise-enhanced neuroplasticity targeting motor and cognitive circuitry in PD.” This article focuses on the improvement of exercise on cognition, neuroprotection, neural recovery, and brain health of patients with PD. In addition, goal-based exercise increases cognitive participation. However, many exercises are aimed at improving balance because balance disorder has a high incidence in patients with PD (Petzinger et al., 2013).

### Analysis of Countries and Institutions

**Figure 4:**
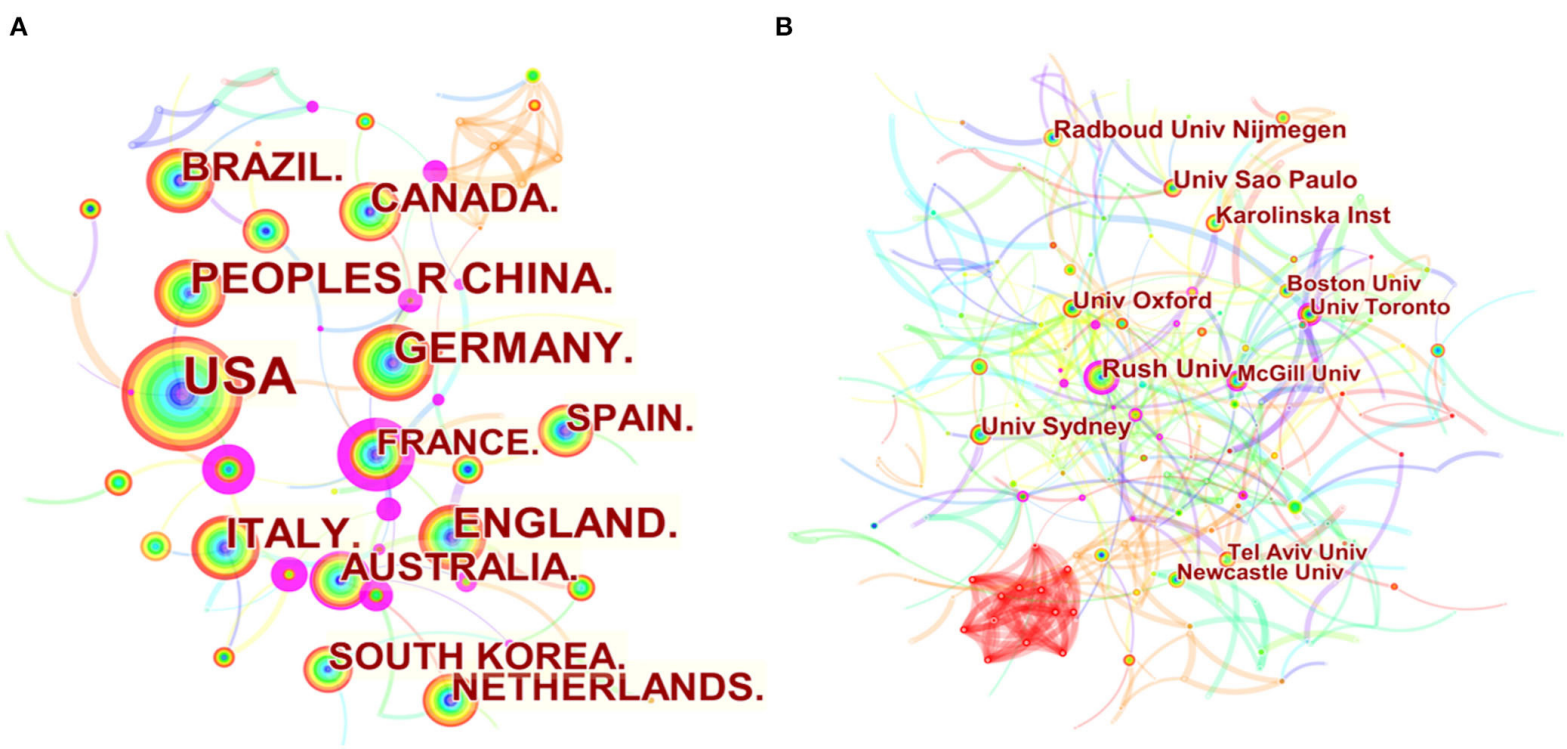
Country and institutional maps of exercise on PD research. **(A)** Map of countries involved in exercise research on PD. **(B)** Map of institutions related to exercise on PD research.

**Figure 5:**
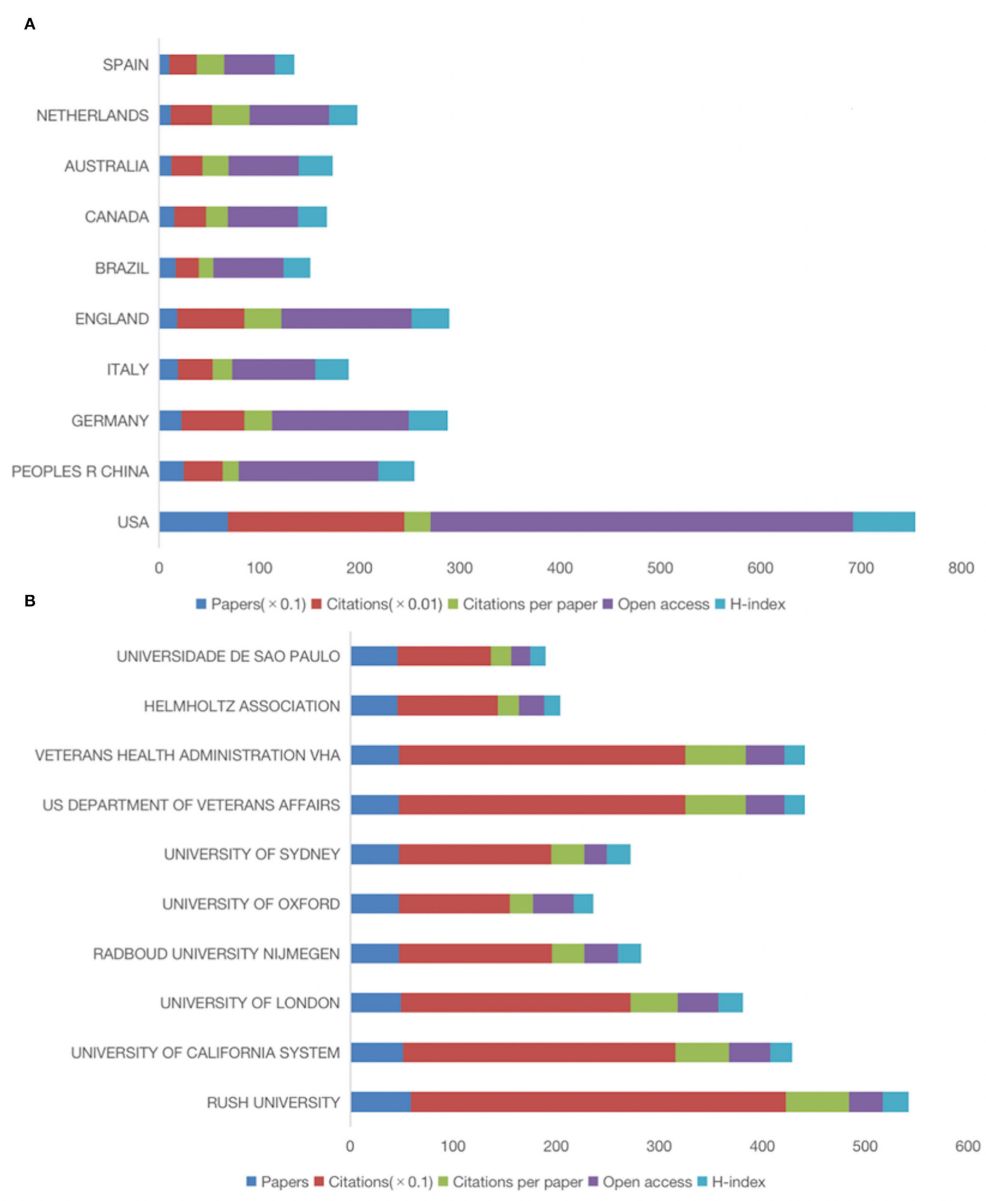
Country and institutional maps of exercise on PD research. **(A)** Number of papers, citations, citations per paper, number of open access papers, and H-index for the top 10 countries. **(B)** Number of papers, citations, citations per paper, number of open access papers, and H-index for the top 10 academic institutions.

The countries and institutions of 2,222 documents searched were analyzed by CiteSpace software, and the country map (Figures 4A, 5A) and the institution map (Figures 4B, 5B) were developed. A total of 76 countries and 2,695 institutions have published such articles, with the top three countries being the United States, China, and Germany. The top three countries in terms of citations are the United States, the England, and Germany. The top three centrality countries are the United States (0.54), the England (0.15), and France (0.12). Centrality indicates that the node establishes a bridge between two unrelated nodes, having a high centrality shows the degree of importance that the node plays in the structure. In CiteSpace, nodes with centrality >0.1 are called critical nodes. The top three countries in the H index are the United States, Germany, and the England. The analysis of the number of articles, citation, centrality, and H index shows that the United States is the most influential country in the field of sports research on PD and maintains close cooperative relations with other countries. The United States has the number of first published articles (684), 17,610 citations, 25.75 citations per article, Open access ranks first (422), and highest H index (62). It plays a leading role in the field of Parkinson's research, and except for the United States, England, France, and Israel cooperate strongly with other countries.

Among the research institutions, the top three institutions in terms of publication volume are Rush University, the University of California System, and University of London. The top three institutions in citation volume are Rush University, US Department of Veterans Affairs, and the Veterans Health Administration. The top three central institutions are Rush University, McGill University, and the University of Toronto, and the top three in the H index are Rush University, University of London, and University of Sydney. According to the number of articles, citation, centrality, and H index, the research on PD by exercise is mainly carried out in universities, whereas Rush University is the main research institution in this field, ranking first in the number of articles (59), citation (3,635), citation per article (61.61), and H index (25). Open access has 33; it plays a leading role in the field of PD, and its main research direction is neurosciences neurology, rehabilitation, and sport sciences. Rush University, McGill University, University of Toronto, Columbia University, Oregon Health, and Science University cooperate closely with other institutions.

### Analysis of Authors

**Figure 6:**
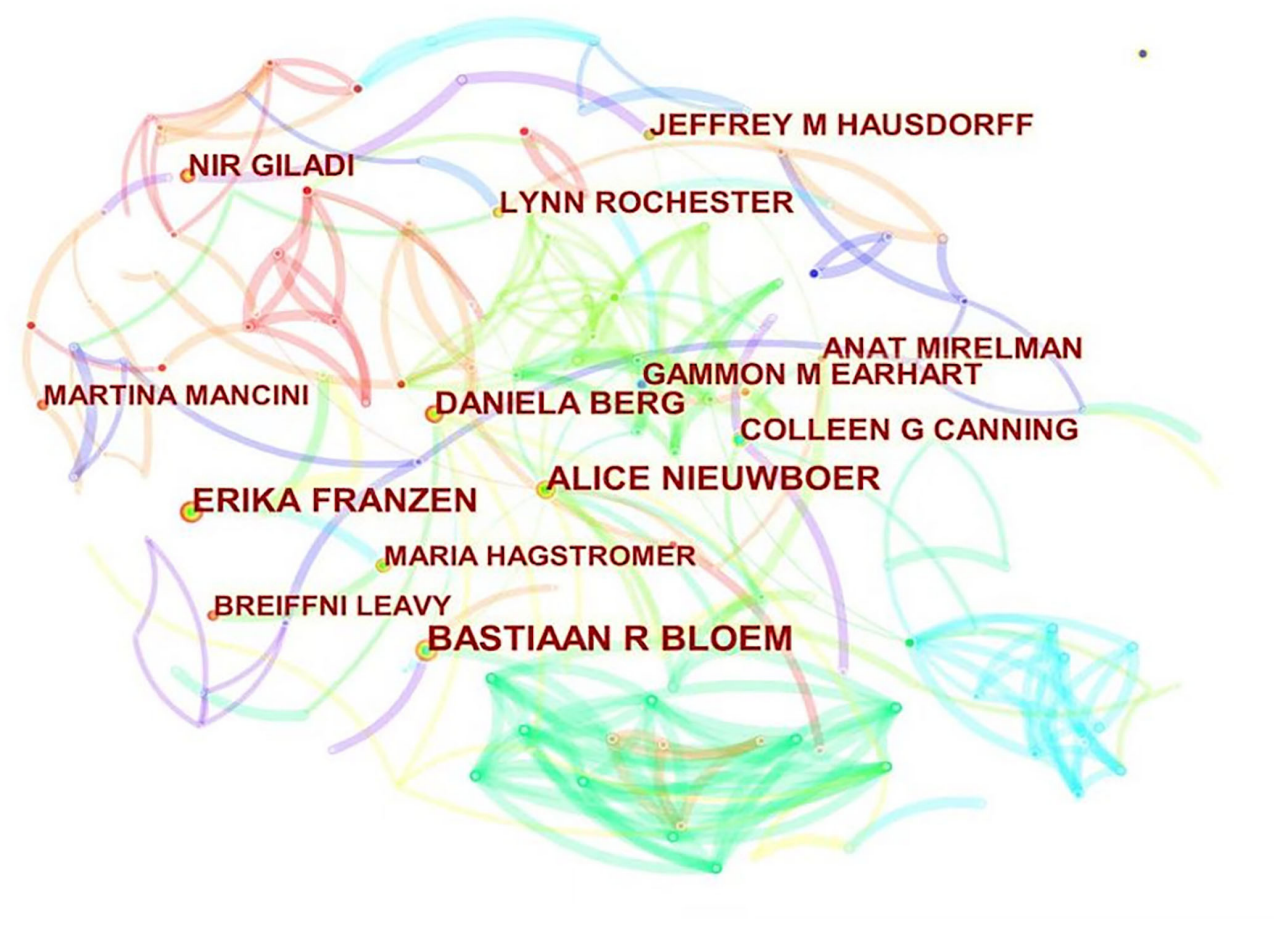
Network graph of the relationship between authors related to the research of exercise on PD.

Among the 2,222 articles searched, 10,046 authors participated in the publication of 546 journals and produced a network graph of related authors (Figure 6). The cooperative relationship between the authors can reflect influential research teams in this research field and provide important information. As shown in Table 1, the top three authors are Bloem BR (39), Franzen E (29), and Nieuwboer A (28). According to the author's centrality, the first three names are Nieuwboer A (0.08), Bloem BR (0.05), and Berg D (0.04). According to the number of posts and centrality, Bloem BR and Nieuwboer A are the main authors in this field, whereas Neurosciences Neurology is the main research field of Bloem BR and Nieuwboer A. The top three cited authors are Goetz CG (464 times), Hoehn MM (403 times), and Hughes AJ (351 times). The most frequently cited paper of Bloem BR is “The Role of the Frontal Lobe in Complex Walking Among Patients With PD and Healthy Older Adults: An fNIRS Study” (133 times). This study proposes that Parkinson's patients can enhance their gait ability by improving neural control efficiency, cognitive ability, and motor ability and emphasize the role of frontal lobe activation in Parkinson's patients. This article provides an important theoretical basis for subsequent studies on PD, and the rapid increase in the number of articles published after 2016 may also be related to this article. This article is the first report of changes in the degree of prefrontal activation during walking in PD patients, which has an important role in gait rehabilitation in PD patients by increasing cognitive control to compensate for deficits during walking (Maidan et al., 2016). Previous studies have demonstrated that exercise has an important role in neuroplasticity in PD patients, and future research studies should focus on the mechanisms of action in prefrontal activation by exercise.

**Table 1:** Top 10 authors, co-citing authors, and co-citing references of exercise on PD.

**Author**	**Published articles**	**Cocited author**	**Cited times**	**Cocited reference**	**Cited times**
Bloem BR	39	Goetz CG	464	The role of the frontal lobe in complex walking among patients with Parkinson's disease and healthy older adults: an fNIRS study	133
Franzen E	29	Hoehn MM	403	The effects of highly challenging balance training in elderly with Parkinson's disease: a randomized controlled trial	100
Nieuwboer A	28	Hughes AJ	351	Feasibility and effects of home-based smartphone-delivered automated feedback training for gait in people with Parkinson's disease: a pilot randomized controlled trial	92
Berg D	24	Tomlinson CL	339	Impaired trunk stability in individuals at high risk for Parkinson's disease	52
Brown P	23	Morris ME	265	Movement-related changes in local and long-range synchronization in Parkinson's disease revealed by simultaneous magnetoencephalography and intracranial recordings	119
Hausdorff JM	23	Fahn S	264	The role of the frontal lobe in complex walking among patients with Parkinson's disease and healthy older adults: an fNIRS study	133
Canning CG	22	Folstein MF	253	Exercise for falls prevention in Parkinson's disease A randomized controlled trial	139
Giladi N	22	Jankovic J	247	The role of the frontal lobe in complex walking among patients with Parkinson's disease and healthy older adults: an fNIRS study	133
Earhart GM	20	Allen NE	200	Barriers to exercise in people with Parkinson's disease	146
Mirelman A	20	Nieuwboer A	200	The role of the frontal lobe in complex walking among patients with Parkinson's disease and healthy older adults: an fNIRS study	133

*WoS, web of science; IF, impact factor*.

### Analysis of Journals

**Figure 7:**
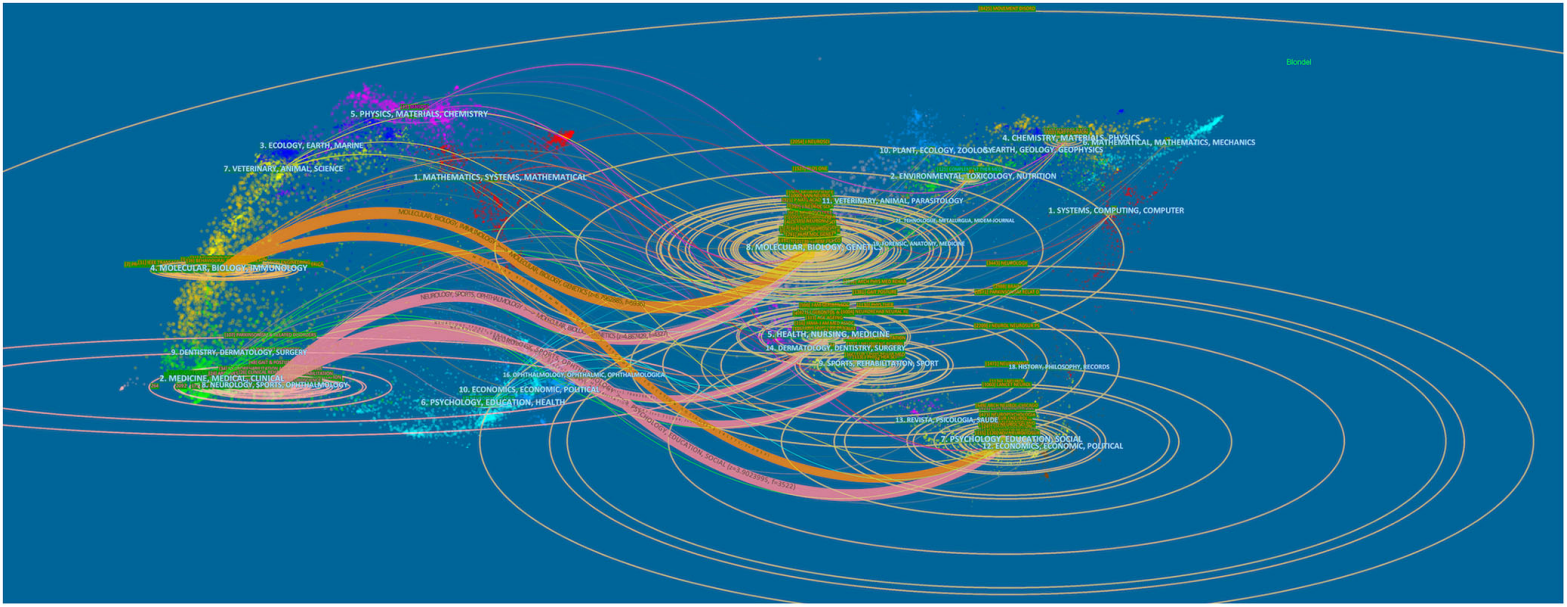
The dual-map overlay of journals related to exercise on PD.

A total of 546 academic journals on exercise on PD have been published, As shown in Table 2, the number of articles published in *Parkinsonism-related Disorders* journals is the largest (107). The number of citations in the journal *Movement Disorders* (5,049) and the number of citations per article (66.43) are the highest, and the journal has the highest impact factor (10.338 in 2020) and the highest H index (33). The number of *PLoS ONE* journals Open access (71) is the highest. Figure 7 shows a double map of periodicals, with cited journals on the left and cited journals on the right. The horizontal axis of the ellipse represents the number of authors, the vertical axis of the ellipse represents the number of published papers, and the curve represents the strength of the citation connection. The atlas shows that the journals with the most contributions are in the fields of neurology, sports, and ophthalmology, and the most frequently cited journals are in the fields of molecular, biology, and genetics.

**Table 2:** Top 10 journals in the field of exercise on PD.

**Journals**	**Papers**	**Citations (WoS)**	**Citations per paper**	**Open access**	**WoS sort**	**IF (2020)**	**Quartile**	**H-index**
Parkinsonism related disorders	107	2,015	18.83	33	Clinical neurology	4.891	Q1	27
Movement disorders	76	5,049	66.43	37	Clinical neurology	10.338	Q1	33
PLoS ONE	71	1,837	25.87	71	Multidisciplinary sciences	3.24	Q2	26
Frontiers in neurology	64	529	8.27	64	Clinical neurology; neurosciences	4.003	Q2	13
Journal of Parkinson's disease	59	702	11.9	30	Neurosciences	5.568	Q1	15
Gait posture	48	1,098	22.88	15	Neurosciences; Orthopedics; Sport sciences	2.84	Q3	18
Parkinson's disease	48	1,179	24.56	48	Clinical neurology	2.704	Q3	15
Neurorehabilitation	37	453	12.24	2	Clinical neurology; Rehabilitation	2.138	Q4	13
Neurorehabilitation and neural repair	34	1,075	31.62	17	Clinical neurology; Rehabilitation	3.919	Q2	16
BMC neurology	29	414	14.28	29	Clinical neurology	2.474	Q3	12

### Features of the 10 Most Frequently Cited Papers

The production table of the top 10 papers cited in sports on PD research is shown in Table 3. The first 10 papers are cited 4,048 times, accounting for 9.26% of the total cited amount (43,728), and the paper with the largest number of citations is the diagnostic criteria for mild cognitive impairment in PD: Movement Disorder Society Task Force guidelines (1,312 times). The journal paper with the highest impact factor is Tai Chi and Postural Stability in Patients with PD (91.253 in 2019). In the first 10 papers, 9 with impact factors >10 were published in the *New England Journal of Medicine, Lancet Neurology, Angewandte Cheme International Edition, Brain*, and *Movement Disorders* according to the ranking of impact factors.

**Table 3:** Top 10 most cited papers on exercise for PD.

**Title**	**First author**	**Journal**	**IF (2019)**	**Year**	**Citations (WoS)**	**WoS sort**
Diagnostic criteria for mild cognitive impairment in Parkinson's disease: movement disorder society task force guidelines	Litvan, I	Movement disorders	10.338	2012	1,312	Clinical neurology
Tai Chi and postural stability in patients with Parkinson's disease	Li, FZ	New England journal of medicine	91.253	2012	429	Medicine, general and internal
Consensus statement on the classification of tremors. from the task force on tremor of the international Parkinson and movement disorder society	Bhatia, KP	Movement disorders	10.338	2018	413	Clinical neurology
How to identify tremor dominant and postural instability/gait difficulty groups with the movement disorder society unified Parkinson's disease rating scale: comparison with the unified Parkinson's disease rating scale	Stebbins, GT	Movement disorders	10.338	2013	362	Clinical neurology
Exercise-enhanced neuroplasticity targeting motor and cognitive circuitry in Parkinson's disease	Petzinger, GM	Lancet neurology	44.182	2013	340	Clinical Neurology
Accuracy of the Microsoft kinect sensor for measuring movement in people with Parkinson's disease	Galna, B	Gait and posture	2.84	2014	304	Neurosciences, Orthopedics, Sport Sciences
International Parkinson and movement disorder society evidence-based medicine review: update on treatments for the motor symptoms of Parkinson's disease	Fox, SH	Movement disorders	10.338	2018	255	Clinical neurology
A redox modulatory Mn3O4 nanozyme with multi-enzyme activity provides efficient cytoprotection to human cells in a Parkinson's disease model	Singh, N	Angewandte chemie-international edition	15.336	2017	220	Chemistry, Multidisciplinary
Opicapone as an adjunct to levodopa in patients with Parkinson's disease and end-of-dose motor fluctuations: a randomized, double-blind, controlled trial	Ferreira, JJ	Lancet neurology	44.182	2016	215	Clinical neurology
Glucocerebrosidase activity in Parkinson's disease with and without GBA mutations	Alcalay, RN	Brain	13.501	2015	198	Clinical neurology, Neurosciences

*WoS, web of science; IF, impact factor*.

### Analysis of References

**Figure 8:**
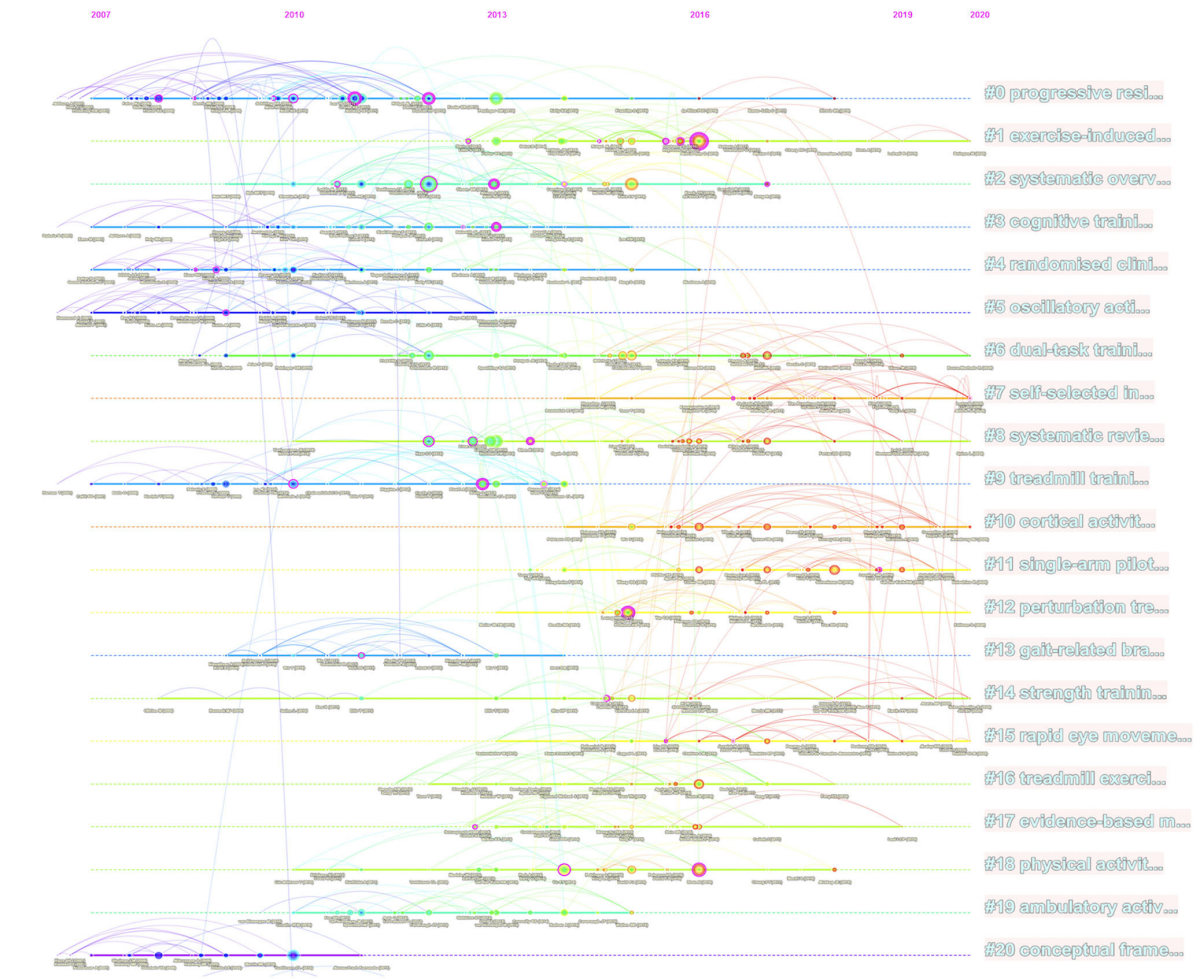
Timeline view map related to exercise on PD.

The analysis of references is an important part of bibliometrics research. Figure 8 shows the top 21 clusters in which references are cited. All clusters are extracted by citing the index words of references. The largest cluster progressive resistance marker is # 0, the second-largest cluster exercise-induced marker is # 1, the third-largest cluster systematic overview marker is # 2, and the fourth largest cluster cognitive training marker is # 3.

### Analysis of Keywords

**Figure 9:**
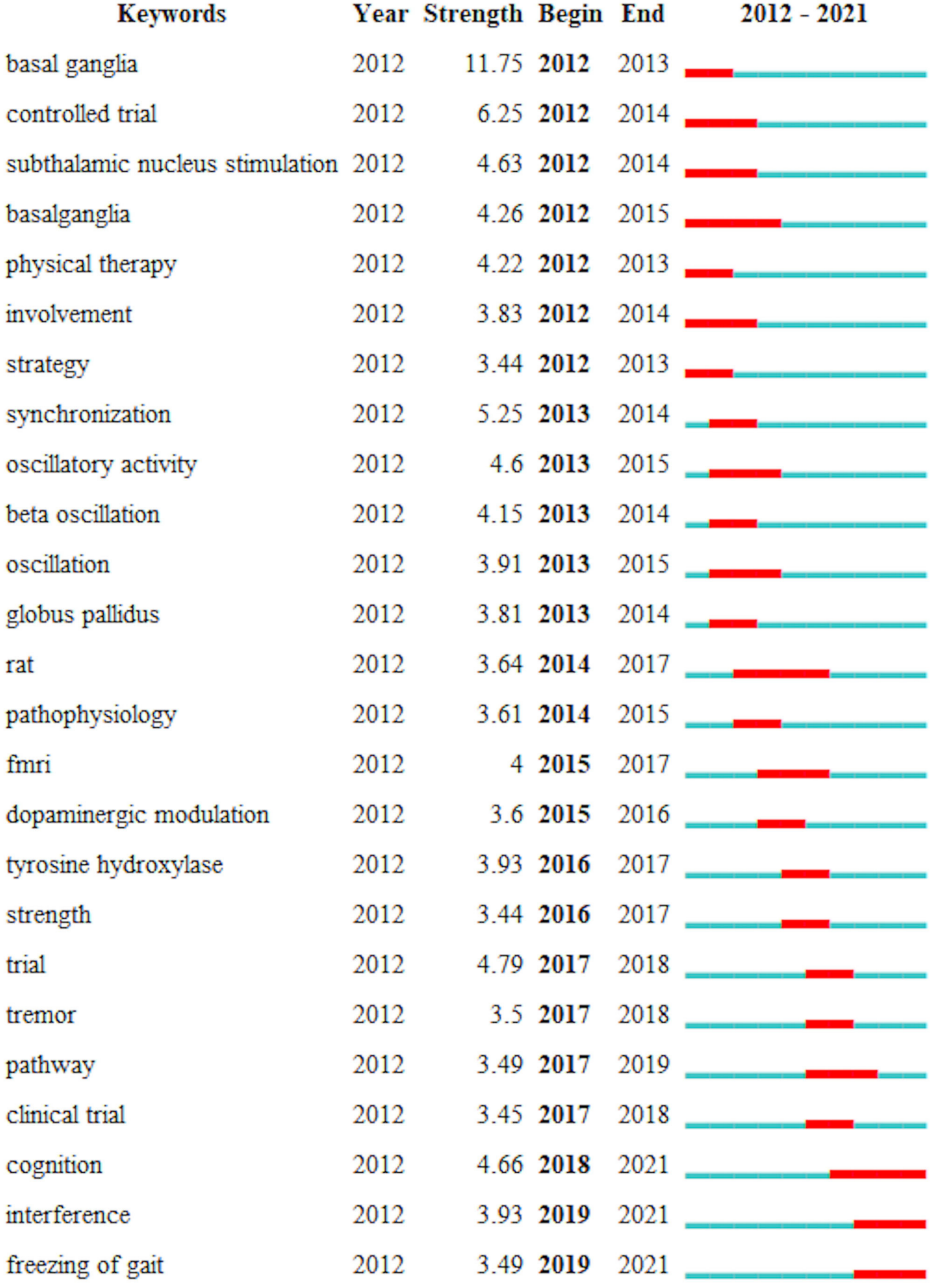
Top 25 key words with the strongest citation bursts. Red bars: some key words cited frequently; blue bars: key words cited infrequently.

The citation degree of keywords reflects the degree of concern of the discipline and can reflect the trend of the development of the discipline. Figure 9 shows the top 25 keywords with the highest cited frequency of exercise to PD in the past decade. The first 25 keywords are analyzed in two dimensions, namely, the burst value and the burst time period. The results obtained through the burst value can determine the trend of the topic over time. Keywords with high burst values imply high research attention and influence in the corresponding time period. Among the 25 keywords, the keyword with the highest burst value is basal ganglia (11.75), and the keywords with the longest burst period are basal ganglia, rat, and cognition. The top three keywords with the highest burst value between 2012 and 2016 are basal ganglia (11.75), controlled trial (6.25), and synchronization (5.25). From 2017 to 2021, the keywords with the highest burst value became trial (4.79), cognition (4.66), and interference (3.93), indicating that the research hotspot and development trend of exercise in the field of PD in recent years have changed to trial, cognition, and interaction In addition, with 2016 as the node, the focus of animal experiments and medical treatment is shifted to human rehabilitation intervention and the transformation of cognitive level.

## Discussion

### Global Research Trends of Exercise on Parkinson's Disease

PD is the second-largest neurodegenerative disease; it is second only to Alzheimer's disease (Wirdefeldt et al., 2011). In a global survey of neurological diseases in 2015, PD caused the fastest increase in disability and mortality (Feigin et al., 2017). PD mainly affects the elderly. The number of PD patients over 50 years old is estimated to increase from 4.1 to 8.7 million from 2005 to 2030, and will be widely concerned by countries all over the world (Dorsey et al., 2007). As an important way to treat PD, exercise therapy has a favorable effect on non-motor disorders in patients with PD (Feng et al., 2020). The analysis of this study is based on the research on PD caused by exercise for 10 years from 2012 to 2021. A total of 2,222 articles were obtained through the retrieval strategy, and the data were imported into CiteSpace software to extract relevant bibliometric indicators (volume of articles, disciplines, countries, institutions, and other indicators) to develop corresponding maps.

This study conducts a systematic bibliometric analysis of exercise-related research on PD in the past 10 years. The results show that the number of articles published has gradually increased in the past decade, especially in the last 5 years (2017–2021), accounting for 61% of the total, indicating that exercise has received more attention in the field of PD in recent years. The years 2012 to 2013 had the highest citation volume (13,986), the highest citation volume per article (43.84), and the highest H index (62). However, the growth in the number of papers in this form does not indicate an improvement in the quality of the literature. This condition is because the retrieval process of literature data extraction only obtains the number of papers in this field, and the quality cannot be judged by the quantity. The high volume of papers and citations can only reflect the attention of the scientific research team in this field. The rapid increase in the publication of papers in the past 5 years may be related to the Guide to American Physical Activity published in 2018, which points out that physical exercise can effectively reduce the risk of many chronic diseases, and that the elderly should engage in various forms of physical exercise, such as balance, aerobics, and resistance exercise (Piercy et al., 2018). In addition, in studies of different ages and populations, the risk of PD was 40% lower in those who exercised at two ages, approximately 35–39 years and at the end of life, than in those who did not participate in exercise at the same age (Paillard et al., 2015). In the assessment of the risk of PD, jobs with higher levels of physical exertion (e.g., construction workers) have a higher prevalence than those with lower levels of physical exertion, such as sedentary people (e.g., teachers; Xu et al., 2010). Future studies should do more to compare the effects of various forms of exercise on people with PD and to follow the effects of these exercises over time to determine exercise treatment options for people with PD at different levels and ages. The influential Bloem BR author in the field wrote the paper The Parkinson Pandemic-A Call to Action published in 2018. PD will increase the global disease burden in the future, and immediate measures are required (Dorsey and Bloem, 2018).

Among the 76 countries in the field of sports research on PD, the United States is the most influential country, with the highest volume of articles, citations, centrality, and H index. Rush University institution has the highest volume of papers, citation, centrality, and H index, and is the main research institution in this field.

Among all the discipline categories of WOS, Neurosciences, Clinical Neurology, Rehabilitation, Sport Sciences, and Multidisciplinary Sciences are mainly concentrated, indicating that the study of exercise on PD focuses on the recovery of the nerve caused by exercise. In other papers on animal experiments, it was demonstrated that exercise may affect activity-dependent processes in the basal ganglia by altering dopaminergic and glutamatergic neurotransmitters and that the effects of exercise at higher exercise intensities may be important in promoting activity-dependent neuroplasticity (Petzinger et al., 2010), and these effects play a role in patients with PD. However, the quality of studies assessing exercise is low and its role in clinical practice is “exploratory” (Fox et al., 2018). The current understanding of neuroprotective mechanisms through exercise in patients with PD comes mainly from animal models (Paillard et al., 2015). Future studies should refine the mechanisms of the effects of exercise on PD patients and translate these findings to humans to explain whether higher-intensity exercise is also effective in PD patients. In previous drug treatment studies, patients with PD still had some underlying symptoms despite the best drug treatment (van der Marck et al., 2014). On the contrary, the patients with PD induced by exercise can be improved after exercise training, and exercise intervention may improve the effectiveness of drug treatment and reduce the deterioration of the patient condition to a certain extent (Mak et al., 2017).

The volume of articles is mainly concentrated in the United States, China, and Germany. Through the analysis of authors and journals, Bloem BR and Nieuwboer A are the main authors in this field; they maintain a high volume of articles and centrality. Among the articles on exercise on PD, Bloem BR, and Nieuwboer A have a randomized controlled trial published in the journal *The Lancet*, which shows that after 6 weeks of treadmill training and 6 weeks of treadmill and VR equipment training the results show a randomized controlled trial. Treadmill and VR equipment training can significantly reduce the risk of falls in the elderly than simple treadmill training (Mirelman et al., 2016). A review of Bloem BR indicates that non-drug interventions, such as strength, balance, and dance exercises are effective, and the field of physiotherapy has made some progress since 2013 and has become a research hotspot (Bloem et al., 2015). Among the top 10 journals, three journals are in Q1, 3 in Q2, 3 in Q3, and 1 in Q4. Only two journals had an impact factor >5. The number of citations in *Movement Disorders* journals (5,049) and the number of citations per article (66.43) are the highest, with the highest impact factor (10.338 in 2020) and the highest H index (33). It is the most influential journal in this field in the United States.

The dual-map overlay (Figure 7), timeline view map (Figure 8), and keyword map (Figure 9), exercise, and other related forms of treatment have always been an important means of treatment for PD. As early as 2007, the American Sports Medical Association and the American Medical Association jointly launched the “Exercise is Medicine” project, which focuses on increasing exercise to promote health. This project strongly promotes the treatment and prevention of chronic diseases through exercise (Sallis, 2009), and exercise is one of the best factors to prevent non-communicable diseases and death. In a 2015 paper, it was shown that in a trial using dual training for balance and gait as a means to improve balance and gait in patients with PD, there was a significant improvement compared to conventional treatment and care in patients with mild to moderate PD. The inclusion of high challenge factors in exercise compared to usual care had an improvement in balance and gait performance in elderly patients with mild to moderate PD and a positive transfer effect in daily activities, suggesting that exercise with high challenge factors is more likely to improve physical activity in elderly patients with mild to moderate PD. In a review of the efficacy of long-term exercise in PD, it was stated that training of external cues (e.g., auditory, visual cues) in PD patients can be used as a compensatory strategy, thus relying on frontal cortical and cerebellar mechanisms to improve movement and that the ameliorative effect of inducing neuroplasticity through exercise in PD patients is further supported in human studies (Mak et al., 2017). Doctors should emphasize the medical role of exercise in the clinic (Eijsvogels and Thompson, 2015). At present, exercise can effectively improve the symptoms of PD, such as tai chi, aerobics, gait, balance, stretching, and resistance training (Li et al., 2012; Pompeu et al., 2012; Corcos et al., 2013; Shulman et al., 2013).

### Strengths and Limitations

Based on WOS data, this study makes a bibliometric analysis of the current situation and trend of exercise research in the field of PD in the past 10 years with CiteSpace and other analysis software. A total of 2,222 articles were retrieved from 546 academic journals in 76 countries. In addition to the analysis of the volume of publications, citations, and the cooperative relationship among countries, institutions, and authors, the classification of disciplines, types of documents, references, and keywords are analyzed.

This research also has certain limitations. The retrieval strategy is limited to the WOS core database. The social science citation retrieval is set to SCI-Expanded, the language is English, and the papers are limited to articles and reviews. These factors may lead to errors in the results, that is, some high-quality and high-impact articles may not have high citations or be late in publication and are ignored, whereas some high-cited articles may not result in hotspots.

## Conclusion

This study analyzes the papers on PD published in the past 10 years and provides a new perspective for research in this field. Although some limitations exist, it fully expounds on the research hotspots and future development trends of exercise in the field of PD research. The analysis results may enable exercise therapy to carry out more research in the field of PD. Sports will receive increasing attention in the field of PD in the future, In conclusion, this study may help researchers to quickly understand the knowledge structure and current hotspots in the field.

## Data Availability Statement

The original contributions presented in the study are included in the article/Supplementary Material, further inquiries can be directed to the corresponding author/s.

## Author Contributions

J-WC wrote the paper. S-HD performed the data analyses. T-CC and KZ contributed to the conception of the study and helped further revise the paper. All authors contributed to the article and approved the submitted version.

## Conflict of Interest

The authors declare that the research was conducted in the absence of any commercial or financial relationships that could be construed as a potential conflict of interest.

## Publisher's Note

All claims expressed in this article are solely those of the authors and do not necessarily represent those of their affiliated organizations, or those of the publisher, the editors and the reviewers. Any product that may be evaluated in this article, or claim that may be made by its manufacturer, is not guaranteed or endorsed by the publisher.
