# Supplementary material for: Research Hotspots and Trends of Exercise on Parkinson's Disease: A Global Bibliometric Analysis From 2012 to 2021
Source: Front Hum Neurosci. 2022 May 27;16:908049. doi: 10.3389/fnhum.2022.908049 (PMC9184738; doi:10.3389/fnhum.2022.908049)
Supplement: Supplementary file 1 [file Table_1.DOCX]

**Supplementary1** Search strategy for Web of Science

#1 TI = (Parkinson's OR Parkinsonism OR Parkinson)

#2 TI = (exercise OR train OR training OR movement OR activity OR activities OR strength OR endurance OR resistance OR stability OR walk* OR tai chi OR yoga OR motor control OR core control OR stretch* OR run* OR muscle energy technique OR Pilates* OR hydrotherapy OR water sports OR kinesiotherapy)

#3 #1 AND #2

Timespan=2012.01.01-2021.12.31 Databases=SCI-EXPANDED. LANGUAGE: (English)

Document type: article and review
